# Neural and endothelial cell-derived extracellular vesicles mediate Zika virus genome dissemination and productive infection *in vivo*

**DOI:** 10.1371/journal.pone.0337609

**Published:** 2025-11-26

**Authors:** María-Angélica Calderón-Peláez, Myriam L. Velandia-Romero, J. Manuel Matiz-González, Jaime E. Castellanos

**Affiliations:** 1 Virology Group, Vice-chancellor of Research, Universidad El Bosque, Bogotá, Colombia; 2 Molecular Genetics and Antimicrobial Resistance Unit, Vice-chancellor of Research, Universidad El Bosque, Bogotá, Colombia; The Scripps Research Institute, UNITED STATES OF AMERICA

## Abstract

Zika virus (ZIKV) is a neurotropic flavivirus linked to severe neurodevelopmental defects following prenatal exposure. While the mechanisms by which ZIKV spreads within the central nervous system remain incompletely understood, extracellular vesicles (EVs) have emerged as potential mediators of intercellular communication and viral dissemination. Here, we demonstrate that EVs derived from ZIKV-infected neural cells encapsulate full-length viral genomes capable of establishing productive *in vivo* infection, independent of free virions. Primary cortical neurons, astrocytes, and mouse brain microvascular endothelial cells (MBECs) from neonatal mice were infected with ZIKV at a low multiplicity of infection (MOI 0.1). EVs were isolated and treated with acid glycine buffer and RNAase to exclude residual virions or free RNA. RNA sequencing, RT-qPCR, and droplet digital PCR (dd-PCR) analyses revealed that EVs—particularly those derived from neurons and MBECs—encapsulated ZIKV RNA, including full-length viral genomes. These EVs were able to transfer viral RNA to A549 cells *in vitro*, and its intracranial injection into neonatal mice resulted in productive infection, confirmed by detection of ZIKV capsid protein, viral RNA, and viral antigen in brain tissue. Our findings demonstrate that EVs from ZIKV-infected neural cells can serve as vehicles for genome transfer and initiate infection, even in the absence of detectable virions. The persistence of EVs-packaged genomes post-viremia could explain clinical observations of prolonged ZIKV RNA within the nervous tissue or delayed transmission. Understanding this pathway provides new insights into ZIKV neuropathogenesis and opens potential avenues for therapeutic intervention, for example targeting EVs biogenesis or cargo sorting.

## Introduction

ZIKV is a member of the Flaviviridae family. It is an enveloped virus with an icosahedral capsid and a single-stranded, positive-sense RNA genome, primarily transmitted by the bite of female *Aedes* mosquitoes [1–3]. Additional transmission routes include blood transfusion, vertical transmission, and sexual contact [4,5]. Approximately 80% of infected individuals remain asymptomatic, while symptomatic cases are generally mild and self-limiting [5]. Severe manifestations occur in about 1% of the cases, with Guillain-Barré Syndrome (the most significant neuropathy in adolescents and adults) being among the most frequently reported complications [6,7], alongside myelitis and meningoencephalitis [8].

These neurological complications highlight ZIKV ability to affect the developing nervous system; however, the mechanisms by which the virus access and disrupts neural tissue remain incompletely understood. One proposed entry route involves viral dissemination via the bloodstream, exploiting the blood–cerebrospinal fluid barrier (B-CSF) [9]. Another hypothesis suggests that ZIKV may utilize circulating monocytes as Trojan horses to cross into the central nervous system (CNS) [10]. Additionally, ZIKV may traverse the blood–brain barrier (BBB) by infecting MBECs, which could release the virus into the brain parenchyma through transcytosis while maintaining BBB integrity [11]. Beyond these mechanisms, ZIKV may also reach the CNS indirectly through alternative pathways, such as via EVs.

EVs are heterogeneous, membrane-bound structures secreted by virtually all cell types under physiological and pathological conditions [3,12,13]. Virus can hijack the EVs biogenesis pathways, resulting in EVs that carry viral particles or genetic material capable of initiating infection upon transfer to uninfected cells, thereby facilitating viral propagation [14]. For instance, EVs from the C6/36 mosquito cell line infected with dengue virus serotype 2 (DENV-2) carry the complete viral genome, which is infectious to human skin keratinocytes and endothelial cells [15,16]. Similarly, hepatitis C virus (HCV), can establish productive infection through EVs harboring its full viral genome, indicating that viral particle assembly is not always required for infectivity [17].

In the context of ZIKV, evidence implicates EVs play a role in placental alterations following infection. Infected macrophages release abundant EVs that interact with human placental trophoblasts, inducing an active inflammatory environment that may enhance ZIKV pathogenicity [18]. This suggests that ZIKV could exploit EVs as molecular shuttles to cross the placental barrier (PB) and subsequently invade the neural tissue. Indeed, EVs efficiently cross both the BBB and the PB, mediating bidirectional communication between the CNS and peripheral tissues [19], supporting the hypothesis that ZIKV may use EVs to disseminate within nervous tissue [3].

Despite these insights, direct evidence for the role of EVs derived from CNS cells in ZIKV pathogenesis remains limited. Therefore, the objective of this study was to determine, using multiple technical approaches, whether EVs released by ZIKV-susceptible nervous tissue cells carry viral RNA, and whether this RNA retains infectious and pathogenic properties. To this end, we isolated and characterized EVs from primary cultures of astrocytes, neurons and MBECs, obtained from one-day-old postnatal (P1) mice infected with ZIKV. Then, we assessed the infectivity of these EVs both *in vitro* and *in vivo*. Our results demonstrate that EVs derived from ZIKV-infected neurons and MBECs (collected at 48 h post-infection, hpi) contain the complete viral genome and are capable of initiating infection in neonatal mice, with viral replication and associated signs of infection observed between 7- and 10-days post inoculation.

## Materials and methods

### Animals and welfare considerations

All experimental procedures were approved by the Ethics Committee of the Universidad El Bosque (Bogotá, Colombia, record No. 013–2019) and the National Institute of Health of Colombia (record No. 11–2019), in compliance with Colombian regulations (Law 84/1989) and the international and Colombian standard guidelines for animal research management.

1. **Housing and husbandry:** Adult Balb/c mice and offspring were housed in a dedicated isolated room of the animal facility of the Universidad Nacional de Colombia, that met all standards for proper care. Ventilated cages were used (500 cm² floor area; NexGen500, Allentown) containing sterile wood chip bedding (Aspen Chip and Lab Grande Aspen, NEPCO). Each cage was equipped with a 250 mL external plastic water bottle and a Whatman filter to allow sterile air exchange and protect food placed in a half-pocket wire bar lid, ensuring adequate nutrition for survival and reproduction. Polycarbonate shelters as environmental enrichment (60 mm × 78 mm) was provided in all cages. Appropriately labeled cages were changed weekly, with litter sizes and birth dates recorded systematically.
2. **Monitoring of animal welfare:** Throughout the experimental period, health and behavior were assessed, focusing on maintenance activities (grooming, feeding), exploratory behavior, social interactions (maternal care, huddling, sexual) and neurological status (posture, mobility). Any abnormality triggered immediate veterinary consultation.
3. **Protocol for the early euthanasia/humane endpoints for animals who became severely ill/moribund during the experiments:**
  - **Predefined termination/euthanasia criteria:** If the animals exhibited signs such as weight loss, poor appetite, impaired movement, visible tumors, or abnormal behavior for more than two days, the veterinarian was informed, and animals were immediately euthanized (by overdose of anesthetic or cervical dislocation).
  - **Once animals reached endpoint criteria, the amount of time elapsed before euthanasia:** 1–2 h maximum
  - **None of the experimental animals died before meeting the criteria for euthanasia.** However, two of the parental animals (that were only used to produce the pups for the experiments but were not used directly in the experiments) presented abnormal behavior. The veterinarian was immediately informed, and the animals were euthanized.
4. **Euthanasia of neonatal mice:** Postnatal pups were euthanized via anesthetic overdose (ketamine and xylazine) in accordance with institutional guidelines.
5. **Other relevant information:**
  - The duration of the experiment was 10 days for the *in vivo* model. The 10-day experimental window for *in vivo* studies balanced developmental neurobiology considerations with ethical constraints.
  - For the *in vitro* model, pups were euthanized at 1 day-old. Cell isolation protocols minimized neonatal sacrifice through optimized cryopreservation (MBECs) and passage strategies (astrocytes).
  - The numbers of animals used were: For *in vivo* model 12 animals, infected at 1 day-old and kept until the 10-day post-infection. *In vitro* cultures were established with cells from 1-day-old pups as follows. Astrocytes: 1 pup per T-75 cm^2^-culture flask. These cells were purified and allowed to replicate. Neurons: 3 pups per experiment. MBECs: 10 pups were used for the entire experiment.
  - How frequently animal health and behavior were monitored: Daily
  - All researchers are trained in the use of animal models for scientific research. Procedures were conducted by researchers (MACP and MLVR) certified by The Colombian Association for the Science and Welfare of the Laboratory Animal, the Institutional Committees for the Care and Use of Animals (CICUA) of the Pontificia Universidad Javeriana and the Universidad de los Andes and the Research Ethics Committee of the National University of Colombia.

### ZIKV harvesting and titration

The study utilized a clinically isolated Colombian ZIKV strain (C11541, GenBank OP898541.1). Viral stocks were generated by initial passage in Vero cells (ATCC CCL-81), followed by three amplification passages in C6/36 HT mosquito cells (ATCC CRL-1660) cultured in L-15 medium (Biowest, L0300) supplemented with non-essential amino acids (MEM NEAA, Gibco 1140−050), 0.3% tryptose phosphate broth (Gibco 18050−039), and 2 mM L-glutamine (Biowest, X0550). After seven days of incubation, supernatants were titrated via plaque assay [20] on BHK-21 cells (ATCC CCL-10), aliquoted and stored at –80°C until use.

### Cell culture isolation

All procedures adhered to the 3Rs principle, using minimal neonatal Balb/C mice (3–5 pups per experiment). Pups were euthanized via ketamine/xylazine (90/15 mg/kg) and decapitated. Brains were dissected for cell isolation as described below.

For astrocytes isolation, tissue was enzymatically dissociated using a mix of collagenase/dispase/DNAse (20 min, 37 °C), as described by [21]. After mechanical dissociation, the tissue was placed on an ovomucoid solution (with BSA, DNAse and Trypsin inhibitor), centrifuged at 1600 rpm and washed with culture medium (DMEM/F12 supplemented with 10% of FBS, L-glutamine, and antibiotic/antimycotic -AA-). Then cells were seeded and incubated with culture medium at 37°C and 5% CO_2._ At 10 days post-seeding, astrocytes were purified by overnight orbital shaking (200 rpm, 37°C) to remove microglia [21]. Cultures were maintained for 8 additional days and dissociated with trypsin-EDTA solution (Biowest) prior to seeding 10.000 cells on round glass coverslips or on 6, 12 or 24 well-plates for experiments.

MBECs were isolated using collagenase/DNAse digestion (1 h, 37 °C) as previously described [21]. Briefly, dissociated brains with no cerebellum were enriched using centrifugation on BSA cushion (1000 rpm −10 min) and Percoll separation to enrich microvessels. These were seeded in 12-well plates with DMEM/F12 supplemented with 3 μg/mL puromycin for 3 days. Cultures were maintained up to 20 days in DMEM/F12 containing 20% FBS, GlutaMAX, AA, heparin (15 U/mL), bFGF (1 ng/mL), and astrocyte-conditioned medium [21].

Cortical neurons were isolated using the protocol from Beaudoin et al [22] using 5 brains per T75 cm^2^ culture flask. Briefly, cortices were digested 20 min at 37°C with trypsin/DNase (0.25%/100 µg), cells were dissociated mechanically and filtered using 70-mesh cell strainer and cleaned via 4% BSA cushion centrifugation. Neurons were plated on poly-L-lysine-coated surfaces in DMEM/F12 with 10% FBS. After 1h of adhesion, cultures were maintained in Neurobasal medium with B-27, L-glutamine, and AA. At 24 h post-seeding glial proliferation was suppressed with 2.5 µM cytosine β-D-arabinofuranoside (AraC) treatment.

### IF cell characterization

Astrocytes (10.000 cells), MBECs (30.000 cells) or neurons (50.000 cells) were seeded on glass coverslips pre-coated with Poly-L-lysine (10 µg/ml for astrocytes and MBECs, and 500 µg/ml for neurons). Twenty-four hours after seeding, cells were fixed with 4% paraformaldehyde (PFA), permeabilized with Triton X-100 (0.3%), and blocked with 10% goat serum. Different primary antibodies were used according the cells: for astrocytes, GFAP (Z0334, Dako) and Glutamine synthase (Santa Cruz, sc-74430); for MBECs, ZO-3 (40–2200, Invitrogen-Thermo Fisher Scientific), and Occludin (Ocln, Cell signaling, 91131), and for neurons CRMP-2 (Cell signaling 35672) and βIII-Tubulin (βIII-Tub, Chemicon, MAB1637) [23]. Secondary conjugated antibodies (Alexa 594 or 488, Invitrogen-Thermo Fisher Scientific) and DAPI (Merck, 10236276001) were applied before mounting with ProLong Gold Antifade Reagent (9071, Cell Signaling). Images were acquired on a Zeiss Axioimager M2 microscope with Colibri 7 fluorescence system and the Apotome 2 deconvolution system (Zeiss) and analyzed using Zen 2.6 software.

### Cell cultures infection and viral detection

Confluent primary cell cultures were infected with ZIKV at a MOI of 0.1 for 1 h at 37°C. Following inoculum removal, cells were maintained in fresh medium for 48 h.

Infection was confirmed via IF assay using the ZIKV capsid-specific antibody (Novus, NBP3–13200) as described before, and absolute RT-qPCR. Total RNA was extracted from cell monolayers and supernatants using TRIzol®. ZIKV RNA copies were evaluated by absolute quantification via RT-qPCR using the probes/primers set previously described [24]. A standard curve was generated from serially diluted ZIKV genomic RNA (BEI Resources, NR-50244) and used to correlate the cycle threshold (Ct) values with the number of RNA molecules per microliter. The number of RNA copies (molecules/μL) was calculated according to the formula: [RNA concentration (μg/mL)]/ [RNA transcript length (nucleotides) × molecular weight of one nucleotide (330 Da) × Avogadro’s number (6.023 × 10²³)]. Thermal cycling parameters: 95 °C for 10 minutes, followed by 40 cycles of 95 °C for 15 seconds and 60 °C for 1 minute in a Biorad CFX96 thermocycler, and processed using the CFX Maestro software.

### Evaluation of infectious viral particles produced in the isolated cultures

A549 and BHK-21 cells (20,000/well in 96-well plates) were exposed to supernatants derived from neuronal, astrocytic, or MBECs infected cultures (diluted 1:1 with fresh culture medium containing 2% FBS). At 24–72 h post infection cells were fixed, permeabilized (0.3% Triton X-100) and endogenous peroxidases were quenched with 50% methanol/0.25% H_2_O_2_. After blocking (10% goat serum), samples were incubated with overnight at 4°C with flavivirus-specific 4G2 antibody (Novus, NBP2–52709). The next day, after washing, cells were incubated with an HRP-conjugated secondary antibody (anti-rabbit 5220–0336 (074–1506), KPL-SeraCare) for 1h at room temperature. Signal detection was performed using a diaminobenzidine and H₂O₂ substrate solution in 0.1 M Tris-HCl (pH 7.2), and staining was analyzed under a Zeiss Axiovert A1 inverted microscope. Images from 2 independent cultures (each with 3 replicates per condition in 8 fields per well) were acquired using Zen 3.1 software (Zeiss). Infected cell counts were performed using the Cell Counter tool (Fiji/ImageJ2), and data is presented as the mean of those counts.

### EVs isolation

For astrocyte and MBECs cultures intended for EVs collection, after removal of the viral inoculum, cells were incubated in UltraCULTURE™ serum-free medium (Lonza, BP12-725F) supplemented with 2 mM L-glutamine (Biowest, X0550). In the case of neuronal cultures, the medium (Neurobasal supplemented with B27) did not require FBS supplementation, which made it particularly suitable for EVs collection. After 48 hpi, EVs isolation was performed as previously described [23]. Briefly, supernatants from IC and their corresponding NIC were collected and centrifuged at 400 *× g* for 15 min and filtered through 0.22 μm membranes (Merck Millipore). Subsequently, the supernatants were centrifuged at 4,000 *× g* to remove larger EVs (apoptotic bodies) and concentrated using Amicon Ultra-15 MWCO 100K filters (Merck Millipore) at 4,000 *× g*. Finally, UC was performed at 100,000 × g (1 h, 4˚C, Beckman Optima MAX-TL) using TLS-55 rotor, k Factor 100.2. The resulting EVs pellets were resuspended in 100 μL of filtered, pyrogen-free PBS 1X [25].

Importantly, prior to UC, only the EVs-IC were treated for 30 min with an equal volume of filtered acid glycine buffer (pH 3.0; 8 g/L NaCl, 0.38 g/L KCl, 0.1 g/L MgCl₂·6H₂O, 0.1 g/L CaCl₂·2H₂O, 7.5 g/L glycine) [26]. This treatment was performed to inactivate free viral particles not enclosed within EVs, as previously described [26,27]. The treated supernatant was then subjected to UC under the aforementioned conditions. The resulting pellet (known as EVs-Gly) was washed with PBS and UC a second time. This pellet was resuspended again in PBS and treated 30 min with RNase A (100 µg/mL, EN0531, Thermo Fisher) to degrade external RNA molecules. The suspension was then UC one final time, and the resulting EVs (referred to as EVs-GlyR) were resuspended in PBS and stored at –80°C until use. All steps were performed under laminar flow to maintain sterility. This protocol ensures isolation of EVs-encapsulated viral genomes free from contaminating virions or extracellular RNA.

### EVs characterization

i) **NTA and DLS:** EVs-NIC, EVs-IC, and EVs-GlyR, were isolated from 10 mL of cell culture supernatant, resuspended in 1 mL of PBS for transportation on dry ice to Cecoltec (Centro Colombiano de Tecnología, Medellín, Colombia). There, EVs analysis was performed under standard conditions (five captures per sample) using a NanoSight LM20 instrument (NanoSight, Amesbury) equipped with a 640 nm laser.
ii) **Western Blot:** Cell lysates (infected and non-infected) were prepared in RIPA buffer, quantified by BCA (Pierce™ Protein Assay Kit, Thermo), and diluted in Laemmli sample buffer. EVs pellets (NIC and GlyR) were directly quantified by BCA and mixed with Laemmli buffer without prior lysis. A total of 10 µg of cellular protein and 30 µg of EVs protein were resolved on 10–12% SDS–PAGE gels at 120 V for 1.3 h and transferred to PVDF membranes at 1.6 A for 3 h. Membranes were blocked with 1% BSA in 0.1% TBST and incubated overnight at 4 °C with primary antibodies against the EVs markers: CD81, Alix and Flotillin-1 (Cell Signaling: 10037, 92880 and 18634). As negative control, Calreticulin (Cell signaling, 12238). After washing, membranes were incubated with HRP-conjugated anti-rabbit secondary antibody (KPL SeraCare) for 1 h at room temperature. Signals were detected using SuperSignal® West Pico and imaged/analyzed with ChemiDoc™ Image Lab Software (Bio-Rad).

### RNA sequencing strategy for tracking ZIKV genome in EVs

To assess whether EVs from ZIKV-infected astrocytes, neurons, or MBECs carry the complete viral genome, a small RNA-seq dataset (~51 nucleotides) was generated from EVs-GlyR. TruSeq Small RNA libraries (Illumina) were prepared from total RNA extracted from EVs-GlyR and EVs-NIC. Quality control was performed using FastQC v0.12.1 and Cutadapt v5.0 [28], with adapter trimming and removal of low-quality regions (Q-score <20). Reads shorter than 30 nucleotides were removed using the -m 30 option to minimize aberrant alignments caused by low-quality or excessively trimmed sequences. Host-derived reads were removed by mapping to the *Mus musculus* genome (i.e., GRCm39, GenBank accession: GCA_000001635.9) using BWA (aln option) [29], and mapped reads were filtered with SAMtools (view -b –f option) [30]. The remaining reads were mapped the ZIKV C11541 genome using BLASTn [31] and visualized in Proksee [32].

To quantify viral genome abundance, the NCPM metric was proposed, adapted from the traditional Count Per Million (CPM) method to account for variable read lengths (30–51 nucleotides) and normalizing read counts based on differences in sequencing depth [33]. This approach was used since the distribution of 30–51 nucleotides reads was not consistent across experiments, therefore the count of reads was replaced with the total number nucleotides, calculated using SAMtools (stats option). This adjustment led to the adoption of the NCPM metric, defined as: NCPM = (mapped viral nucleotides × 10⁶)/ total number of sequenced nucleotides.

### Quantification of viral transcripts and Western blot for viral protein detection in EVs

Viral genome copies per microliter in EVs-IC and their derivatives (EVs-Gly and EVs-GlyR) were quantified using ddPCR. Total RNA was extracted directly from the EVs samples using the miRVana™ miRNA Isolation Kit (Invitrogen). Each reaction, 5 µL of RNA, 400 nM probe/primer mix targeting the ZIKV envelope (E) gene (HEX-labeled), and the One-Step RT-ddPCR Advanced Kit for Probes (Bio-Rad, 1864021), following the recommended protocol. Reverse transcription and cDNA amplification were performed on a CFX96 thermocycler (Bio-Rad) and droplets were analyzed using the QX200 Droplet Reader (Bio-Rad) using QX Manager software.

For viral protein detection, 10 µL of EVs-pellets were used for protein quantification using the BCA assay. The remaining pellet was mixed with Laemmli buffer, and 30 µg of total protein were separated on a 15% SDS-PAGE gel and transferred to a PVDF membrane at 1.6 A for 3 h. Membranes were blocked with 1% BSA in TBST (0.1%) and incubated overnight at 4°C with the ZIKV capsid antibody (1:1000 dilution) under gentle agitation. After washing, membranes were incubated with HRP-conjugated anti-rabbit secondary antibody for 1h at room temperature. Detection was performed using the SuperSignal® West Pico Chemiluminescent substrate, and images were acquired and analyzed with the ChemiDoc™ Image Lab Software (BIO-RAD).

### Evaluation of infectivity of EVs-GlyR *in vitro* and *in vivo*

For *in vitro* assays, 10,000 A549 cells per well were seeded into 96-well plates. After 24 h, the cells were inoculated with 3 × 10⁶ EVs (NIC, IC, or GlyR) derived from the various primary cell cultures, corresponding to a ratio of approximately 300 EVs per cell. Cultures were then incubated for 72 h at 37 °C in 5% CO₂. Following this period, some wells were fixed with 4% PFA and processed by immunoperoxidase staining, using the ZIKV capsid antibody (Novus, NBP3–13200). Remaining monolayers were pooled, resuspended in Trizol®, and total RNA was extracted for absolute RT-qPCR detection of viral transcripts, as previously described.

For *in vivo* experiments, P1 Balb/c mice were anesthetized with local lidocaine and ice, then inoculated i.c. with 3 x10^6^ EVs-NIC or EVs-GlyR (n = 4 per condition). Control groups included animals inoculated with mock (supernatants from non-infected C6/36 cells, n = 4), and mice inoculated with 400,000 PFU/mL of ZIKV (n = 4). Mice were monitored daily for 10 days post-inoculation), with body weight and size recorded every 24 h. At 10 days post-inoculation, mice were anesthetized with ketamine (15 mg/kg) and xylazine (5 mg/kg), perfused intracardially with 15 mL PBS, and whole brains were collected and sectioned.

The left hemisphere was fixed in 4% PFA at 4 °C for 48 h, embedded in paraffin, sectioned at 5 µm, and mounted on poly-L-lysine–coated slides. Sections were deparaffinized, rehydrated, and subjected to antigen retrieval prior IF staining for ZIKV capsid detection (Novus), astrocytes (GFAP, Dako), and neurons (MAP2, Cell Signaling, 8707). Imaging was performed using a Zeiss Axio Imager M2 fluorescence microscope equipped with Colibri 7 illumination system and Zen 2.6 software. The right hemisphere was divided for molecular analyses: one portion was homogenized in TRIzol® for RT-qPCR detection of ZIKV transcripts, and the other in RIPA buffer for Western blot assessment of viral antigen.

### ZIKV detection in whole brain lysates by Western blot

Brain homogenates were lysed in RIPA buffer for 30 min on ice, centrifuged at 14000 x *g* at 4°C, and supernatants were collected for protein quantification by BCA assay. Fifteen micrograms of total protein were separated by 15% SDS-PAGE gel, transferred to a PVDF membrane and probed with ZIKV capsid antibody (Novus, NBP3–13200). Signal detection was performed as described above using the ChemiDoc™ Imaging System (BIO-RAD).

### Statistical analysis

All experiments were conducted in triplicate and repeated in two or three independent experiments. Mock-infected cells and animals (supernatants from non-infected C6/36-HT mosquito cells) served as controls. Data normality was assessed using the Shapiro-Wilk test (p > 0.05). Statistical significance was determined using ANOVA or Kruskall-Wallis (p < 0.05), followed by Dunnet’s or Dunn multiple comparison *post-hoc* tests. Unpaired t-tests (p < 0.05) were employed for specific comparisons of viral RNA copy numbers. Significance thresholds were set at *p < 0.05, **p < 0.01, ***p < 0.001, and ****p < 0.0001.

## Results

### Neurons, astrocytes, and MBECs cultures show differences in susceptibility and production of infectious virions

**Fig 1:**
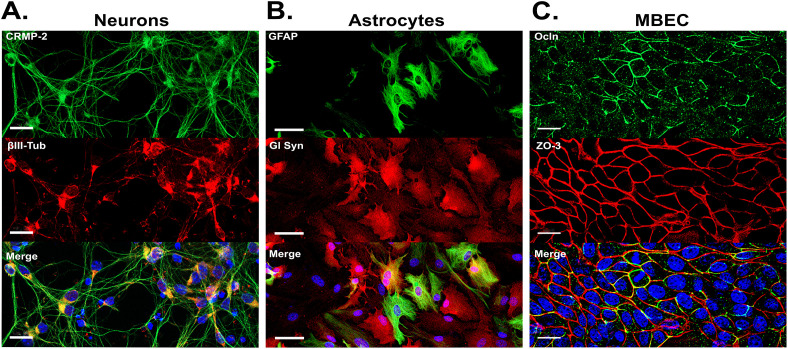
Characterization of primary neuronal, astrocytic, and MBECs cultures. (A) Neuronal cultures exhibited robust expressions of βIII-tubulin and CRMP-2 markers, indicative of differentiated neurons undergoing structural and functional maturation. (B) Astrocyte cultures co-expressed Gln Syn and GFAP consistent with a mature and metabolically active astrocytic population. (C) In MBECs cultures, the co-localization of tight junction proteins ZO-3 and Ocln demonstrated the formation of a well-differentiated endothelial monolayer with intact barrier properties. Nuclei were counterstained with DAPI (blue). Scale bars represent 20 µm for neurons and MBECs and 50 µm for astrocytes. Representative images from three independent experiments, each consisting of three replicates with eight fields captured per replicate.

Primary cultures of cortical neurons, astrocytes, and MBECs were isolated from P1 mice and maintained *in vitro* for 7–20 days depending on the specific requirements of each cell type. Cell populations reached 95–98% purity, confirmed by immunofluorescence (IF) assays and morphological criteria (Fig 1), guaranteeing experimental responses could be attributed to a single cell type. Cortical neurons exhibited small soma with extensive neurite arborization, positive for CRMP-2 and βIII-tubulin (Fig 1A). Astrocytes displayed a stellate morphology with hypertrophic processes, and co-expressed GFAP and glutamine synthase (Gl Syn), reflecting functional metabolic activity and indicating heterogeneous astrocyte population (Fig 1B). MBECs cultures formed confluent monolayers with polygonal morphology, with compact cytoplasm and well-defined intercellular junctions, stained for ZO-3 and occludin (Ocln), consistent with intact barrier properties (Fig 1C).

**Fig 2:**
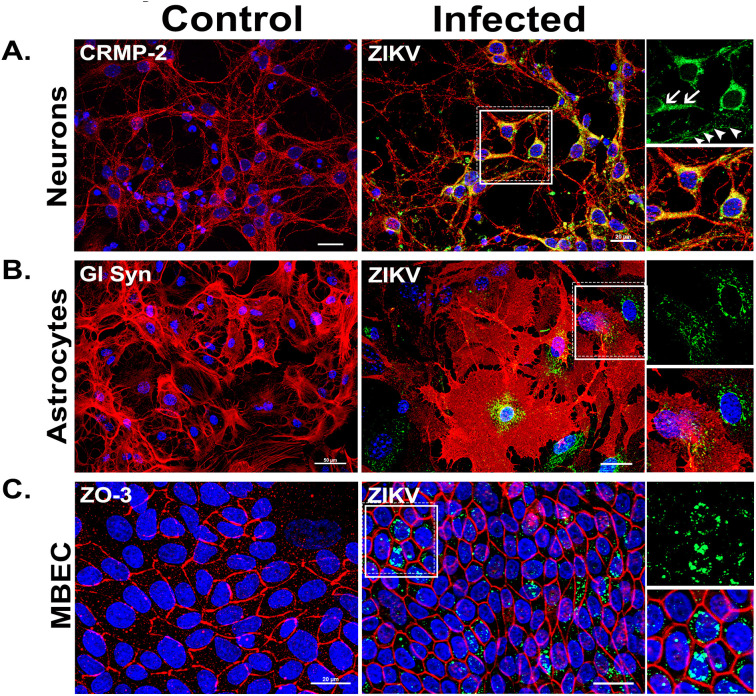
Detection of ZIKV capsid “C” protein in neuronal, astrocytic, and MBECs cultures. Infection of all three cell types was confirmed by IF detection of the ZIKV C protein (green) at 48 hpi. Infected cells were identified through co-staining with cell-type specific markers (red). While all cultures demonstrated susceptibility to ZIKV infection, (A) neurons exhibited the most pronounced morphological alteration compared to uninfected controls. Notably, viral antigen is localized not only to perinuclear region but also to the axon hillock (arrow) and neurites (arrowhead). In contrast, (B) astrocytes and (C) MBECs maintained their typical cellular architecture despite infection. The highlighted square denotes a region of interest shown at higher magnification. Nuclei were counterstained with DAPI (blue). Scale bars represent 20 µm for neurons and MBECs, and 50 µm for astrocytes. Images are representative of two independent experiments, each comprising three replicates and eight fields analyzed per slide.

Cultures were inoculated with ZIKV at MOI of 0.1 for 1 h. At 48 hpi, IF revealed distinct infection patterns across cell types (Fig 2). Neurons exhibited pronounced susceptibility, with viral capsid protein localized to perinuclear regions, axon hillocks (arrows), and distal neurites (arrowheads), suggesting axonal trafficking (Fig 2A). Astrocytes showed moderate infection, marked by cellular hypertrophy with small perinuclear accumulation of viral antigen (highlighted boxes, Fig 2B), while MBECs demonstrated reduced cell size (Fig 2C).

**Fig 3:**
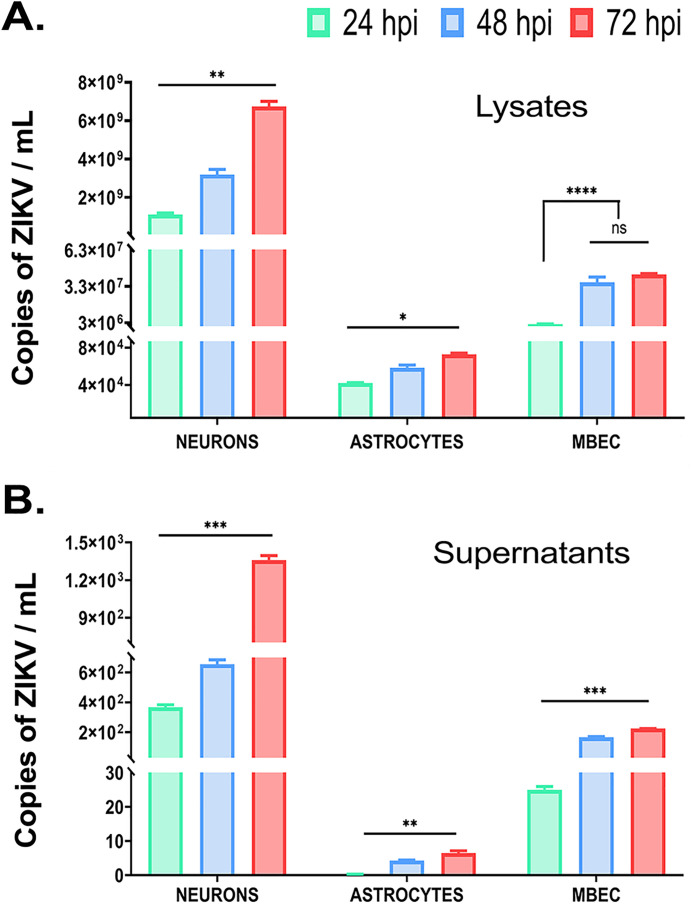
Quantification of ZIKV viral transcripts. Viral RNAs were extracted from both (A) lysates (reflecting intracellular viral load) and (B) culture supernatants (representing extracellular free virus) of primary neurons, astrocytes and MBECs at indicated times post-infection. Absolute RT-qPCR was performed to quantify ZIKV transcripts, with results expressed as “ZIKV copies/mL”. Across all cell types and time points, intracellular viral copy numbers were consistently higher than those detected in the supernatant. Among the three cell types, neurons exhibited the highest levels of both intracellular transcript production and extracellular viral release. Data represents the mean of three independent experiments each performed in duplicate. Statistical analysis was performed using one-way ANOVA followed by Dunnet’s multiple comparison test: *p < 0.05, **p < 0.01, ***p < 0.001, ****p < 0.0001).

Intracellular and extracellular viral RNA levels were quantified via RT-qPCR at 24, 48, and 72 hpi (Fig 3). Neuronal lysates contained the highest intracellular RNA copies by 24 hpi (1.1x10^9^ copies/ml), increasing to 6.74x10^9^ copies/ml by 72 hpi (Fig 3A). Corresponding supernatants mirrored this trend, peaking at 1.3x10^3^ copies/ml of viral RNA at 72 hpi (Fig 3B). MBECs exhibited delayed replication, with intracellular RNA plateauing at 48 hpi (3.63 copies/ml RNA) and supernatant titters stabilizing by 72 hpi with 2x10^2^ copies/ml RNA (Fig 3A, B). Astrocytes showed minimal viral RNA intracellularly (< 10 copies/ml RNA, Fig 3A) and undetectable extracellular viral titters at 24 hpi, rising marginally to 6.5 copies/ml of viral RNA by 72 hpi (Fig 3B), suggesting either reduced susceptibility or a more effective antiviral response in these cells.

Finally, the infectivity of viral particles present in the supernatants of primary neuronal, astrocytic, and MBECs cultures was assessed at 24, 48, and 72 hpi. Supernatants collected at each time point were used to inoculate BHK-21 cells. Following a brief adsorption period, the inoculum was removed and replaced with fresh medium containing 2% fetal bovine serum (FBS). Cells were then incubated for an additional 72 h before fixation. Viral antigen was detected by immunoperoxidase staining, and the number of infected cells was quantified. As controls, non-infected cells (NIC), mock-inoculated cells and ZIKV (MOI 0.1) were evaluated (S1A Fig).

Consistent with previous observations, supernatants from neuronal cultures yielded robust infection in BHK-21 cells as early as 24 hpi, demonstrating efficient production and release of infectious virions (S1B Fig). MBECs derived supernatants also contained infectious particles detectable at 24 hpi, although the number of infected BHK-21 cells was lower compared to those exposed to neuronal supernatants (S1B Fig). In contrast, supernatants from astrocyte cultures showed no infectivity at 24 hpi, and only minimal infection at 48 and 72 hpi (S1B Fig). These results confirm that neurons, astrocytes, and MBECs are all permissive to ZIKV infection and support viral replication, as evidenced by increasing intracellular viral RNA levels. However, the comparatively low levels of viral RNA in the supernatants suggest that viral release may be tightly regulated, potentially reflecting viral strategies to evade immune activation, prolong cellular infection, or limit dissemination within the CNS.

### ZIKV infection altered both the size and quantity of EVs produced in neurons, astrocytes, and MBECs cultures

To investigate the effects of ZIKV infection on EVs production and size, supernatants from the various ZIKV-infected cultures (MOI 0.1) and their non-infected cells (NIC) were collected at 48 hpi. EVs were isolated by ultracentrifugation (UC) and analyzed by multiple complementary techniques.

**Fig 4:**
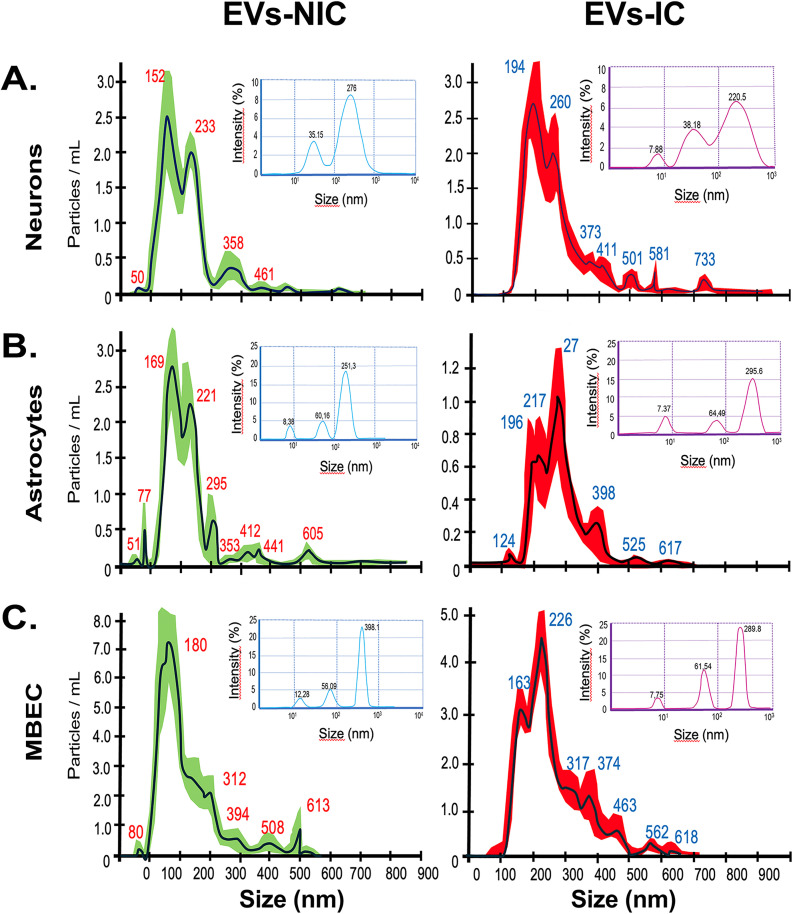
Nanoparticle Tracking Analysis (NTA) and Dynamic Light Scattering (DLS) analysis of EVs from non-infected (NIC) and ZIKV-infected cultures. The smaller insets depict light scattering intensity profiles obtained from DLS measurements, while the larger panels present NTA data, illustrating particle concentration peaks (particles/mL) as a function of particle size (diameter) for each analyzed sample from (A) neurons, (B) astrocytes and (C) MBECs. These analyzes demonstrated that ZIKV infection altered both the size distribution and abundance of EVs produced in each culture compared to their respective NIC. Representative data from two independent experiments.

EVs size and concentration were further characterized by Dynamic Light Scattering (DLS) and Nanoparticle Tracking Analysis -NTA- (Fig 4). DLS intensity plots displayed multiple peaks (insets in Fig 4), indicating heterogeneous populations of particles with varying sizes based on their Brownian motion. Peaks with high intensity percentages corresponded to particle sizes that were highly abundant and contributed substantially to the overall signal detected.

DLS measurements showed a consistent reduction in the average particle size (Z-Average) in EVs derived from infected cultures relative to controls. For example, neuronal EVs (Fig 4A) exhibited a decrease in mean size from approximately 105.4 nm observed in EVs from non-infected cultures (EVs-NIC) to 65.63 nm in EVs from infected cultures (EVs-IC). This size reduction was accompanied by an increase in the polydispersity index (PdI) from 0.589 to 0.892, indicating greater heterogeneity in EVs populations under infection (Table 1). Similar trends were observed in EVs from astrocytes (Fig 4B) and MBECs (Fig 4C), suggesting that ZIKV infection broadly influences EVs biogenesis and release dynamics.

**Table 1:** DLS analysis of EVs from different cell cultures.

Cell Type	Condition	Average Size (Z-Average, d.nm)	PdI	Intercept
**Neurons**	**EVs-NIC**	105.4	0.589	0.929
	**EVs-IC**	65.63	0.892	0.914
**Astrocytes**	**EVs-NIC**	302.8	0.3086	0.9524
	**EVs-IC**	287.4	0.3594	0.9199
**MBECs**	**EVs-NIC**	253.5	0.6221	1.01
	**EVs-IC**	128.7	0.72	1.04

The PdI indicates the uniformity of nanoparticle size within the sample. A PdI close to 0.1 suggests a narrow size distribution (monodispersity), whereas values above 0.3 indicate greater variability in particle size (polydispersity). The intercept value is related to the intensity of the detected signal and reflects the quality of the correlation in DLS. An intercept close to 1 indicates a high-quality signal, meaning that the obtained data are reliable for interpreting particle size distribution.

Astrocyte-derived EVs were generally larger and exhibited a modest size reduction upon ZIKV infection, decreasing from 302.8 nm to 287.4 nm. This change was accompanied by a slight PdI increase from 0.3086 to 0.3594, indicating a limited increase in sample heterogeneity. Similarly, EVs isolated from MBECs showed a minor size decrease from 253.5 nm to 240.9 nm, and a PdI increase from 0.6221 to 0.7552 following infection. Intercept values ranging from 0.91 to 1.01 confirmed the quality and reliability of these measurements, supporting the robustness of the particle size and heterogeneity of data obtained.

Collectively, these findings highlight the heterogeneous nature of EVs populations. However, because DLS assesses the average Brownian motion of all particles simultaneously, its interpretation becomes challenging in polydisperse samples such as EVs. To address this limitation, complementary analysis by NTA was performed to provide a more detailed particle size distribution and to quantify particle concentrations.

NTA results (Fig 4) revealed size distribution of EVs based on particle diameter (x-axis) and concentration (y-axis), further confirming the heterogeneity within EVs populations. Most particles were distributed within the 150–300 nm size range (Fig 4, Table 2). Across all cell types, EVs-IC were slightly larger than their non-infected counterparts, although the average size differences among the three cell populations were modest (Table 2).

**Table 2:** NTA analysis of EVs from all cell cultures.

Cell Type	Condition	Average size (nm)	Mode (nm)	Particles/ml
**Neurons**	**EVs-NIC**	217.3	158.8	3.50e + 08 + /- 2.26e + 07
	**EVs-IC**	263.6	193.8	4.00e + 08 + /- 4.87e + 07
**Astrocytes**	**EVs-NIC**	222.5	168.1	3.24e + 08 + /- 3.50e + 07
	**EVs-IC**	279.7	276.6	1.26e + 08 + /- 1.64e + 07
**MBECs**	**EVs-NIC**	245.1	179.5	9.63e + 08 + /- 1.47e + 08
	**EVs-IC**	261.3	225.4	1.40e + 09 + /- 6.57e + 08

The results show the average size, mode, and particle concentration, along with standard deviations for each EVs group from the different cell cultures.

Regarding particle concentration, neurons released approximately 350 million EVs/mL under non-infected conditions, which increased modestly to 400 million EVs/mL following ZIKV infection. Similarly, MBECs exhibited a pronounced elevation in EVs production, increasing from 963 million EVs/mL in NIC to 1.4 billion EVs/mL upon infection, indicating that viral infection enhances EVs release in these cell types (Table 2). Notably, MBECs were the most prolific EVs producers among the analyzed cell populations. In contrast, astrocytes demonstrated a significant decrease in EVs release following infection, with NIC releasing 324 million EVs/mL compared to only 126 million EVs/mL in infected cultures (Table 2).

These results suggest that ZIKV infection selectively impairs EVs biogenesis and/or release specifically in astrocytes, while promoting EVs production in neurons and MBECs. The data reveals that ZIKV infection differentially modulates both the size and quantitative characteristics of EVs populations in a cell type-dependent manner. This complexity underscores the necessity of employing complementary analytical techniques to achieve accurate and comprehensive characterization of EVs.

Western blot analysis for EVs characterization showed that calreticulin was present in all cell lysates but absent from all EVs preparations, confirming the purity of the isolated vesicles (S2 Fig). Neuron-derived EVs contained Alix, Flotillin-1, and CD81. Astrocyte- and MBEC-derived EVs also expressed these markers; however, Alix levels were reduced in astrocyte-derived EVs-GlyR, and a similar decrease was observed in MBEC-derived EVs-NIC. In contrast, MBEC-derived EVs-GlyR exhibited increased CD81 expression compared with EVs-NIC from the same cells (S2 Fig). This variation likely reflects the distinct EVs protein profiles characteristic of each cell type and experimental condition. Importantly, the detection of canonical EVs markers across samples confirms its successful isolation.

### EVs from ZIKV-infected neurons and MBECs carry viral transcripts

Consistent with observations in other viral models, EVs produced by infected cells (IC) may harbor viral RNA. To assess this possibility in our model, we implemented a stringent protocol to eliminate free virions potentially co-precipitated with EVs. For this, pellets were treated with glycine buffer (pH 3.0) for 15 min, followed by RNAse A digestion after a second round of UC (referred to as EVs-GlyR). This dual treatment ensured that residual free virions and external RNA were inactivated, ensuring that any subsequent infection was attributable solely to infectious material carried within the EVs.

NTA analysis of EVs-GlyR revealed no detectable changes in their size profile compared with EVs-IC. For instance, EVs-IC from MBECs exhibited a mode of 225.4 nm (mean: 261.3 nm), while EVs-GlyR from the same cells showed a mode of 225.7 nm (mean: 260.8 nm), S3 Fig. This indicates that the EVs maintained essentially the same size signature regardless of GlyR treatment. However, a reduction (~51%) in particle concentration was observed in EVs-GlyR. Although we cannot completely rule out the effect of glycine itself, the additional UC steps required to remove residual glycine are the most likely cause of particle loss. Importantly, despite this decrease, the final yield remained sufficient for all downstream experiments.

**Fig 5:**
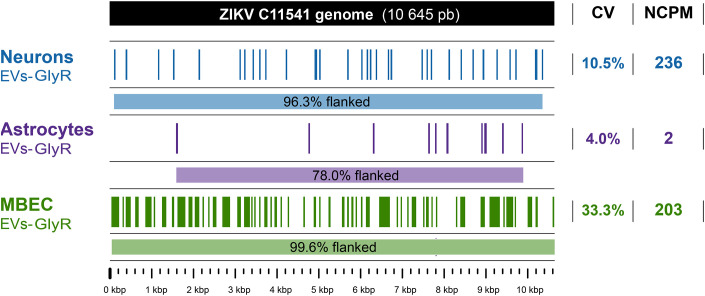
ZIKV C11541 genome was transported as cargo of EVs-GlyR from neurons, astrocytes, and MBECs. For each cell type, the upper panel displays a coverage map of ZIKV C11541 genomic regions detected in EVs-GlyR via reference-based assembly of small RNA-seq data. The lower panel illustrates the full-length viral genome (10.8 kb), with blue, purple or green segments indicating regions flanked by sequenced reads. Metrics include percentage genome coverage (CV) and normalized viral nucleotide counts per million total nucleotides (NCPM). No ZIKV sequences were detected in EVs-NIC. To calculate NCPM, we considered as total the number of nucleotides remaining after excluding low-quality and host reads (Neurons: 16’553,501; Astrocytes: 498’011,206; MBECs: 45’213,763). Viral nucleotides were identified as those within this filtered dataset that aligned with the ZIKV C11541 genome (Neurons: 3,907; Astrocytes: 754; MBECs: 9,196). Scale bar: 1 kb. Representative data from three independent experiments.

To identify molecular features indicative of the presence of the full-length ZIKV genome inside the EVs, a reference-based reconstruction of the viral genome was performed using small RNA-seq data obtained from the EVs-GlyR derived from neurons, astrocytes and MBECs. In all three cases, assembled viral contigs were aligned across the viral genome in a distributed pattern, collectively flanking between 78.0% and 99.6% of its length, despite exhibiting low overall genome coverage -neurons: 10.5%; astrocytes: 4.0%; MBECs: 33.3%- (Fig 5). Additionally, a higher number of viral reads was detected in neurons (NCPM = 236), followed by MBECs (NCPM = 203), and, to a much lesser extent in astrocytes (Fig 5), indicating the presence of viral genetic material in all three cell types but with marked differences in abundance, notably lower in astrocytes. No viral sequences were detected in EVs-NIC controls, confirming infection-specific origin of these transcripts.

Taken together, these results demonstrate that EVs-GlyR carried viral RNA reads exclusively associated with the infected condition, covering a substantial portion of the ZIKV C11547 isolate genome. This supports the notion that EVs-GlyR may transport the complete ZIKV genome, with notably lower representation in astrocytes compared to neurons and MBECs.

**Fig 6:**
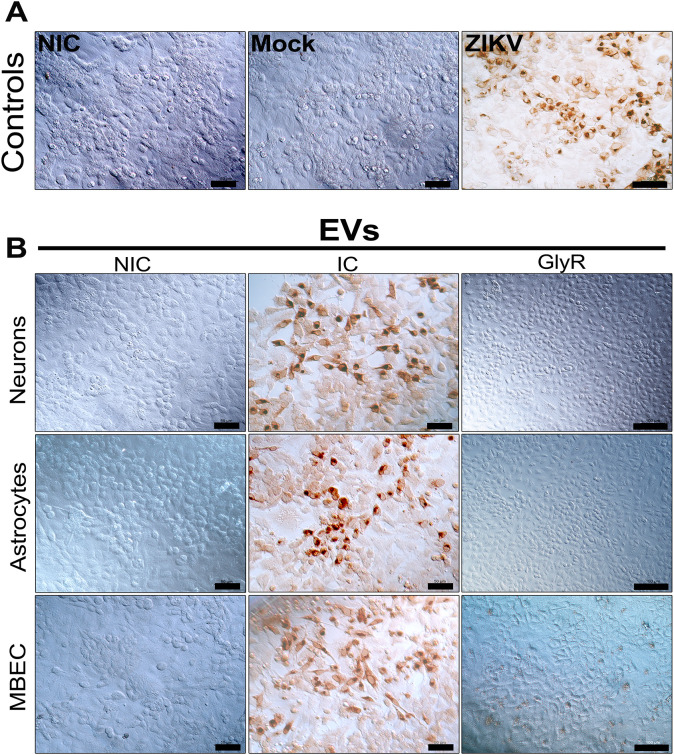
Detection of ZIKV in A549 cells inoculated with EVs derived from infected cultures. Immunoperoxidase staining for ZIKV E protein in A549 cell inoculated for 72 h with EVs or ZIKV. (A) Controls included non-infected cells (NIC), mock-treated cells (supernatants from uninfected C6/36HT cells), and supernatants of ZIKV-infected cells at a MOI of 0.1. (B) Brown peroxidase signal indicated productive infection in cells exposed to EVs-IC, while no detectable staining was observed in EVs-GlyR-treated cells. Representative images from three independent experiments, each performed in triplicate. Scale bars: 100 µm for controls and EVs-GlyR) and 50 µm for EVs-IC.

To evaluate the functional relevance of EVs-encapsulated RNA, A549 cells were inoculated with 3 million EVs-GlyR for 72 h and analyzed via immunoperoxidase staining for ZIKV envelope “E” protein. As controls, NIC, mock-inoculated and ZIKV-infected (MOI 0.1) were included (Fig 6A). While EVs-IC from all cell types induced E protein detection in recipient cells, confirming the co-precipitation of viral particles during UC (Fig 6B), notably, this signal was abolished in EVs-GlyR-treated samples, indicating successful inactivation of free virions (Fig 6B). The absence of detectable infection suggests that EVs-associated viral RNA, though present, either lacks infectivity under these conditions or exists at levels below the assay detection.

Additionally, Western blot analysis of EVs-GlyR pellets revealed detectable ZIKV capsid “C” protein in astrocyte-derived EVs, while E protein remained undetectable across all cell types (S4A Fig). Parallel RT-PCR screening of EVs lysates for contiguous ZIKV genomic regions failed to yield amplicons (S4B Fig), likely due to the limited sensitivity of PCR when analyzing low-copy RNA within EVs. These findings underscore both the heterogeneity of viral protein incorporation across EVs populations and the technical challenges inherent in detecting scarce viral genomes within EVs preparations using standard molecular techniques.

**Fig 7:**
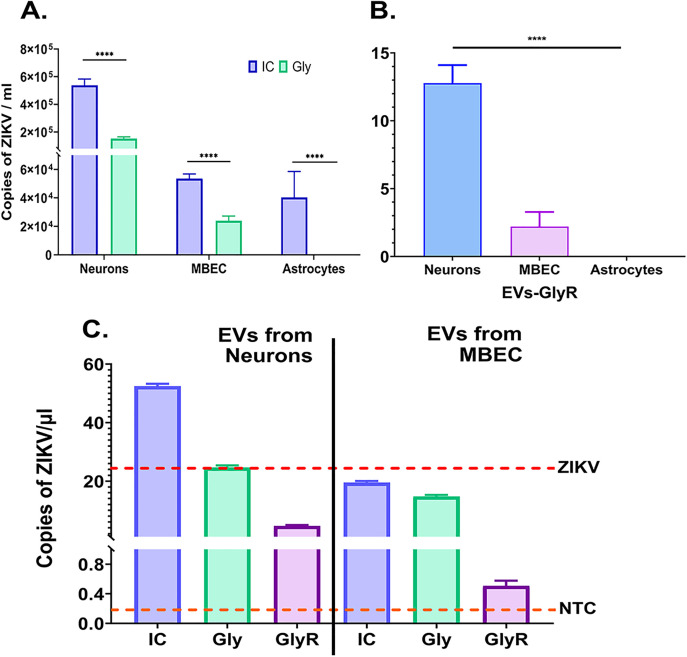
Detection of ZIKV RNA in A549 cells and EVs derived from infected neurons, astrocytes, and MBECs. A549 monolayers were treated with EVs-Gly or EVs-GlyR. (A) Absolute quantification by RT-qPCR revealed detectable viral RNA in cells treated with EVs-Gly. Statistical analysis was performed using a Mann-Whitney test. (B) Only cells treated with EVs-GlyR derived from neurons or MBECs contained measurable ZIKV RNA, whereas no viral RNA were detected in cells treated with EVs-Gly or GlyR from astrocytes. Statistical analysis was performed using a one-way ANOVA followed by Dunnett’s multiple comparisons test as the post hoc test. (C) ddPCR quantified ZIKV RNA copies directly within EVs preparations (EVs-IC, EVs-Gly, and EVs-GlyR) isolated from infected neurons and MBECs. Dotted lines indicate the ZIKV-positive control (virus diluted 1:10,000, red) and EVs from non-infected control (EVs-NIC, Orange). Data represents the mean of two independent experiments performed in duplicate. Statistical significance: ****p < 0.0001.

To enhance detection sensitivity, A549 cells were incubated with glycine-treated EVs (EVs-Gly) or glycine/RNAse-treated EVs (EVs-GlyR) for 72 h. Total RNA extracted from these cells was analyzed via absolute RT-qPCR, to enhance sensitivity by allowing even low‐copy ZIKV genomes delivered via EVs to be taken up, reverse-transcribed, and detected within a cellular context, thereby overcoming the detection limits of direct EVs lysate analysis. Cell exposed to EVs-IC exhibited high viral transcripts levels, consistent with free virions contamination, while EVs-Gly exposed cultures, showed reduced ZIKV RNA copies (Fig 7A). Notably, low but detectable viral RNA was observed in A549 cells treated with neuron- and MBECs-derived EVs-GlyR, with neuronal EVs demonstrating the highest signal (Fig 7B). In contrast, no viral transcripts were detected in cells treated with either EVs-Gly or EVs-GlyR from astrocytes (Fig 7A, B). The detection of viral transcripts in A549 cells suggested that EVs-GlyR from neurons and MBECs seemed to transfer viral RNA. These findings led to the selection of EVs from neurons (EVs-N) and MBECs (EVs-EC) for subsequent analyses.

ddPCR confirmed the presence of ZIKV RNA in EVs-GlyR from neurons and MBECs cultures, with glycine/RNAse treatment reducing RNA copy numbers (Fig 7C). This validate that UC coprecipitates free virions or RNA, which are partially removed by inactivation protocols. Overall, these findings demonstrate that ZIKV-infected neurons and MBECs release EVs containing low levels of ZIKV RNA, even after rigorous virion exclusion. While insufficient to be detected under standard conditions, EVs-mediated RNA transfer may amplify neural damage by facilitating viral genome dissemination within CNS. This mechanism could contribute to ZIKV neuropathogenesis by enabling stealth viral spread independent of free virion production.

### EVs from neurons and MBECs infected, caused brain infection in neonatal mice

Building on evidence from both *in silico* analysis and ddPCR, we evaluated the infectious potential of the EVs by intracerebrally (i.c.) inoculating neonatal mice. A total of three million EVs-NIC or EVs-GlyR derived from neuronal or MBECs cultures were injected into P1 Balb/c mice. Animals were monitored daily for changes in body weight and size. Phenotypic alterations became apparent only after 7 days post-inoculation. Mice receiving EVs-GlyR, irrespective of their neuronal (S5A Fig) or MBECs (S5B Fig) origin, exhibited reduced body size and weight compared to those inoculated with EVs-NIC or mock controls (S5 Fig). Notably, these changes resembled those observed in ZIKV-infected mice (S5 Fig).

**Fig 8:**
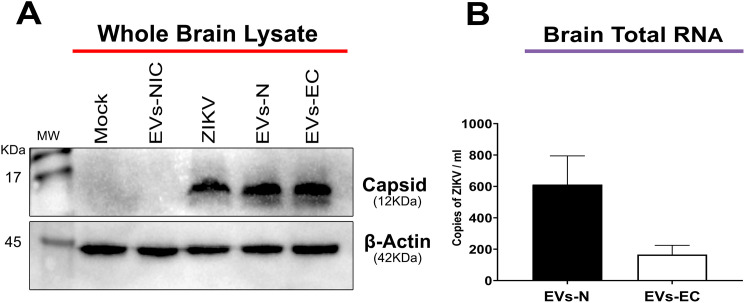
Detection of ZIKV RNA and C protein in brain-lysates from EVs-GlyR inoculated mice. Brains from EVs-inoculated mice were analyzed to assess ZIKV infection. (A) Western blot analysis of whole brain lysates confirmed ZIKV C protein (~12 kDa) in mice inoculated with EVs- GlyR derived from neurons (EVs-N) or MBECs (EVs-EC), as well as in ZIKV-infected positive controls. C protein signal was absent in mock-treated, or EVs-NIC–inoculated mice. β-actin (~42 KDa) served as loading control. (B) ZIKV RNA copies quantified by RT-qPCR in whole-brain lysates revealed significantly higher viral RNA loads in mice receiving neuron-derived EVs-GlyR compared to MBECs-derived EVs-GlyR. Representative data from three independent experiments performed in duplicate. Statistical analysis was performed using a Mann-Whitney test (p < 0.05 threshold), but no significant differences were detected.

**Fig 9:**
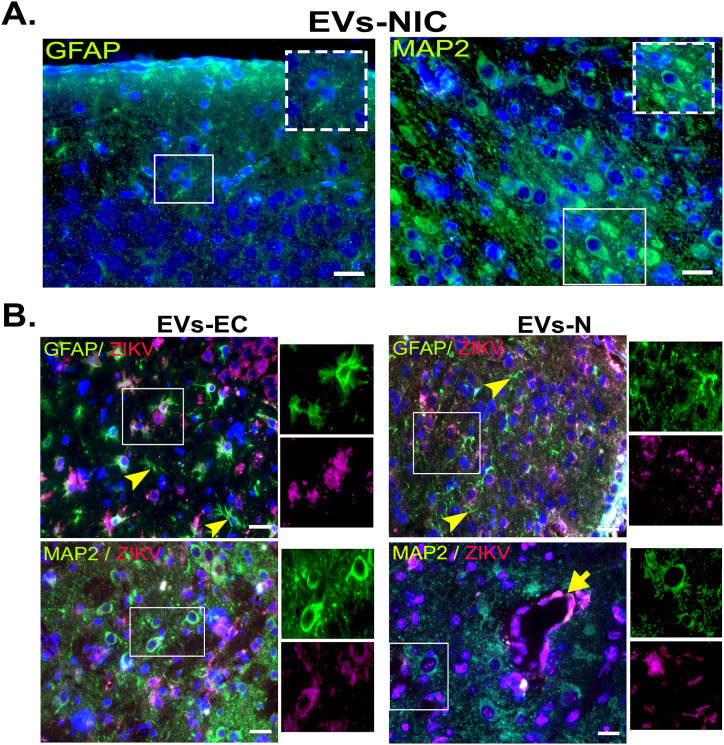
ZIKV virus detection in brain sections from EVs-inoculated mice. IF analysis localized ZIKV C protein (red) in MAP2-positive neurons and GFAP-positive astrocytes in brains of EVs-GlyR-inoculated mice. Activated astrocytes (arrowheads) and ZIKV antigen in capillary-associated endothelial cells (arrows) were observed across all treatment groups. Scale bar: 20 µm. Images are representative of four inoculated animals per group.

At day 10 post-inoculation, when the neurological symptoms such as tremors and ataxia manifested, animals were anesthetized and perfused. Brains were harvested for viral RNA quantification by RT-qPCR, and viral antigen detection by Western blot and IF. Western blot analysis confirmed the presence of ZIKV C protein in whole-brain lysates from animals inoculated with EVs-GlyR (Fig 8A). As expected, strong capsid signals were observed in brains from ZIKV-infected mice (positive control), while no signal was detected in animals inoculated with EVs-NIC or in mock-inoculated groups (Fig 8A). Correspondingly, RT-qPCR revealed 611.5 and 165.6 ZIKV RNA copies/mL in brains of mice inoculated with EVs-GlyR from neurons (N) and MBECs (EC) respectively, indicating successful viral replication initiated by EVs-delivered genomes *in vivo* (Fig 8B). IF analysis localized ZIKV antigen primarily within cortical neurons, albeit with low signal intensity, and in brain MBECs lining capillaries (indicated by arrows, Fig 9). Viral antigen was also detected in a subset of astrocytes. Importantly, reactive astrocytes exhibiting stellate morphology were observed across multiple brain regions regardless of EVs source (arrowheads), consistent with reactive gliosis potentially induced by viral infection or inflammatory responses (Fig 9).

Collectively, these findings provide strong evidence that EVs derived from ZIKV-infected neurons and MBECs carry the complete viral genome capable of initiating infection withing the nervous tissue. Upon internalization by CNS cells, EVs-encapsulated viral RNA appears sufficient to support viral replication and assembly of infectious particles. This study highlights a mechanism by which ZIKV exploits EVs-mediated intracellular communication to facilitate viral dissemination, replication, and pathogenesis within the CNS.

## Discussion

EVs are lipid bilayer-enclosed structures produced by virtually all cell types, varying widely in size and molecular cargo. These structures are released into the extracellular environment under both physiological and pathological conditions, carrying bioactive molecules that modulate recipient cell functions [3,12,13]. During viral infections, EVs can encapsulate and transfer viral RNA, proteins, or even intact virions, thereby evading immune detection and potentially modulating host immune response to facilitate viral dissemination. In the case of neurotropic viruses such as ZIKV, EVs may play a critical role in promoting viral spread and entry into the neural tissues [3].

Despite this potential, direct evidence supporting the involvement of EVs derived from ZIKV-infected neural cells in CNS infection remains limited. In this study, we utilized primary cultures of astrocytes, neurons, and MBECs from neonatal mice to investigate the capacity of EVs released during ZIKV infection (MOI 0.1) to carry and transmit viral material. EVs were isolated and characterized using multiple approaches, including RNA-seq and ddPCR, revealing the presence of viral RNA, including sequences covering the full genome, particularly in EVs derived from MBECs. Functional assays demonstrated that EVs derived from neurons and MBECs not only transferred viral RNA to recipient cells *in vitro* but also initiated infection *in vivo* following i.c. inoculation in neonatal mice. Viral replication and associated neuropathological changes were detected at 10 dpi, as confirmed by RT-qPCR, IF and Western blot analyses. These findings strongly suggest that ZIKV exploits EVs released by infected neural cells to facilitate viral spread and replication within the CNS.

### EVs secreted by some nervous tissue cells were modified in quantity and size during ZIKV infection

Consistent with previous reports, our model confirms that most cellular components of the BBB, a selective interface primarily composed of MBECs in coordination with astrocytes, microglia, pericytes, and neurons [11], are susceptible to ZIKV infection. The BBB is considered a major route for viral entry into the CNS. Infection of endothelial cells in the retina and placenta has been documented [34–36], and our data corroborate that MBECs are highly permissive to ZIKV replication and release, without compromising monolayer integrity, in agreement with prior studies [37].

Astrocytes in our model also exhibited susceptibility to ZIKV infection, consistent with previous reports [38], and showed marked GFAP overexpression together with pronounced morphological alterations. However, unlike studies using human astrocytes that describe early and robust viral replication [38,39], primary astrocytes derived from neonatal mice displayed delayed and limited production of infectious particles. These differences likely reflect species-specific and model-dependent variability, as noted in comparative analyses of human astrocyte lines [40]. Notably, the restricted infection and modest viral output observed here are consistent with the ability of astrocytes to maintain a constitutive antiviral state and to mount rapid innate responses, particularly through early type I IFN signaling and induction of interferon stimulated genes (ISGs). This basal antiviral activity can limit viral assembly and release, often resulting in abortive or non-productive infections in which viral RNA and structural components are produced but the formation and egress of infectious virions remain inefficient [41]. Importantly, this intrinsic antiviral environment, together with the relative resistance of astrocytes to virus induced apoptosis, has been shown to permit prolonged low-level infection and sustained viral shedding, supporting the notion that astrocytes may serve as reservoirs in which ZIKV can persist despite limited productive replication [42]

Furthermore, primary cortical neurons from neonatal mice were highly susceptible to ZIKV infection, supporting robust viral replication and particle release. This highlights ZIKV’s ability to infect not only neural progenitor cells (NPCs) and immature neurons but also postnatally developing neuronal precursors destined to become cortical neurons [43–45].

Viral infections commonly alter host EVs production. Infected cells secrete vesicles containing viral RNAs, proteins, and modified host factors; these EVs often differ in size and abundance compared to those from uninfected cells [46,47]. For instance, untreated patients with human immunodeficiency virus (HIV) produce plasma EVs that are both larger and more numerous than those in uninfected or virologically controlled individuals [47]. Similarly, chronic HCV infection enhances EVs secretion as an innate immune escape mechanism, with EVs enriched in viral RNA and immune-modulatory cargo [48]. Building on this, our results demonstrate that ZIKV infection modifies EVs size distribution, consistent with previous studies indicating changes in vesicle biogenesis and cargo composition may influence viral pathogenesis and cell targeting [49].

Comprehensive characterization of EVs using multiple complementary techniques is essential, as recommended by the MISEV2018 and 2023 guidelines [50,51]. Notably, we observed discrepancies between DLS and NTA in measuring particle size, reflecting inherent methodological differences. The modest size increase detected by NTA (which analyzes particle motion and provides accurate size distributions for smaller vesicles) [52], likely indicates the emergence of a subpopulation of larger EVs during infection. In contrast, DLS measures scattered light intensity and is biased toward larger particles due to its dependence on scattering, potentially overlooking or differently interpreting these subpopulations [53].

### EVs secreted by some nervous tissue cells carry ZIKV proteins and RNA

Viral infections often exploit EVs biogenesis pathways to enhance replication, evade immune detection, and facilitate dissemination by incorporating viral components such as proteins, RNA fragments, or complete virions into EVs, thereby modulating infection dynamics [54]. Given that EVs are produced by nearly all cell types, viral preparation typically represents a mixture of virions and EVs. Disentangling their respective roles requires rigorous separation, a technical challenge due to overlapping size and density profiles [55]. To address this, stringent post-UC purification steps are necessary to remove co-precipitated virions and ensure that downstream analyses reflect the specific authentic EVs cargo rather than residual viral contaminants.

Low-pH glycine-rich buffers have been successfully used for flavivirus inactivation [27,56] leveraging glycine’s zwitterionic properties, which help to preserve protein and membrane integrity more effectively than stronger acids such as citrate or acetate [57]. However, low-pH exposure can subtly alter EVs surface proteins or membrane structure depending on conditions such as pH, exposure time and temperature, and may be less effective against smaller viruses [57]. Although a reduction in EVs yield was observed after treatment and we cannot completely rule out a contribution of the acidic conditions to this outcome, repeated ultracentrifugation and additional wash or pelleting steps required after GlyR treatment are well documented to decrease particle recovery and can even compromise vesicle integrity [58,59]. Empirical assessments further show that each extra wash step tends to trade purity for reduced yield [60,61]. Therefore, careful optimization is essential to balance efficient viral inactivation with preservation of EVs biological function. In our study, a standardized low pH glycine buffer protocol effectively neutralized residual free viral particles without altering EVs size, as evidenced by stable particle distributions in NTA analysis.

Following confirmation of EVs preservation, we treated EVs-IC with acid glycine buffer and RNAse A and assessed the presence of ZIKV RNA using RNA-seq, RT-qPCR, and ddPCR. The RNA-seq analysis revealed viral genomic reads within the vesicular cargo, with coverage spanning 78% to 99.6% of the viral genome. However, due to short length of sequenced fragments (30–51 nucleotides) and the high abundance of small mouse RNAs in EVs, complete viral genome reconstruction was not feasible. To overcome these limitations, long-read sequencing technologies such as Oxford Nanopore technology could generate sufficiently long reads to detect full viral genomes [62]. Additionally, enrichment strategies like genome-tiling PCR, followed by targeted sequencing may enhance recovery of viral material from low-input EVs samples [63].

While RNA-seq suggested that EVs may transport the complete ZIKV genome, further validation was required. Our ddPCR assays on EVs-GlyR confirmed the presence of ZIKV RNA, albeit in low abundance. To further assess their infectivity, EVs-GlyR were inoculated into A549 cells, a highly permissive cell line for ZIKV. Viral RNA was detected in recipient cells at 72 h post-inoculation, indicating successful delivery of viral RNA by EVs. However, immunoperoxidase assays at this time point did not confirm formation of complete viral particles.

Given these inconclusive *in vitro* results, we assessed the infectious potential of neuron- and MBECs-derived EVs-GlyR *in vivo* by i.c inoculation in neonatal mice. Productive infection was established within 10 days post-inoculation, as demonstrated by IF and Western blot detection of ZIKV C protein in whole brain lysates. Importantly, since only astrocyte-derived EVs-GlyR contained detectable C protein directly, its presence in brains inoculated with neuron- and MBECs-derived EVs implies de novo viral replication. The absence of C protein in neuron- and MBECs-derived EVs-GlyR further suggests that these EVs do not carry assembled virions. This interpretation aligns with the genomic organization of ZIKV, a non-segmented RNA virus encoding a single polyprotein from a unique open reading frame. Viral replication requires intact 5′ and 3′ untranslated regions for polymerase recognition [64,65], thus, only EVs containing the full-length genome can serve as functional templates for infection, whereas fragmented RNA lacking critical regulatory elements cannot initiate replication. Collectively, these data demonstrate that EVs from infected neurons and MBECs deliver the complete ZIKV genome to susceptible brain cells, enabling polyprotein translation, viral replication, and assembly of infectious progeny.

Our findings contrast with those of Zhao et al. [66], who reported that EVs from ZIKV-infected human umbilical vein endothelial cells (HUVECs) contained high levels of viral E protein and RNA but failed to confer infectivity to recipient cells. This discrepancy likely reflects differences in cellular origin, species, and even in ZIKV strains, underscoring the critical influence of cell type and tissue context on EVs-mediated ZIKV transmission. The neural microenvironment particularly in developing neonatal mouse brain characterized by an immature BBB and permissive immune milieu [67,68], may provide favorable conditions for EVs entry and viral genome delivery, translation, and replication.

On the other hand, our results showed that astrocyte-derived EVs transported the ZIKV capsid (C) protein. This may reflect the strong, cell-autonomous type I interferon response of astrocytes, which can restrict productive flavivirus replication and promote abortive or incomplete replication cycles [41]. Under these conditions, structural proteins may still be translated, but virion assembly and egress remain inefficient. As a result, excess or mislocalized C protein might accumulate and potentially be redirected to multivesicular bodies or secretory-autophagy pathways for disposal or signaling. Moreover, the intrinsic membrane and RNA binding properties of the C protein may make it more susceptible to being captured by EVs biogenesis pathways, whereas the selective incorporation of full-length viral genomes into EVs likely requires specific RNA protein interactions that may be less efficient during restricted infection [69,70]. Altogether, these findings suggest that astrocyte derived EVs may preferentially incorporate the capsid protein while excluding the complete viral genome, consistent with selective sorting mechanisms operating under a partially restrictive antiviral state.

### EVs, another route for ZIKV transmission

Viral transport via EVs has been extensively documented, with EVs serving as vehicles for viral propagation and immune evasion [3]. Within the Flaviviridae family, HCV packages its full genome and the E2 protein into EVs, forming infectious pseudo-viral particles that enhance recipient cell susceptibility [15,71,72]. Similarly, DENV-infected mosquito cells release EVs containing complete viral genome capable of infecting human skin keratinocytes and endothelial cells [16]. Japanese encephalitis virus (JEV)-infected microglia secrete EVs that induce neuronal apoptosis via caspase activation, while EVs carrying non-replicative JEV or DENV subgenomic replicons transfer viral RNA to diverse cell types illustrating an alternative dissemination pathway independent of the classical receptor-mediated entry [73].

Our *in vivo* experiments demonstrate that EVs derived from ZIKV infected neonatal cortical neurons and MBECs carry replication-competent viral genomes. These findings align with prior studies showing that ZIKV-infected embryonic mouse cortical neurons release EVs containing infectious viral RNA [74]. Notably, our work extends this observation to postnatal cortical neurons, highlighting ZIKV’s ability to exploit developmentally mature neuronal populations for EVs-mediated dissemination. Furthermore, we show that a low MOI (0.1) suffices for packaging full-length viral genomes into EVs, suggesting this mechanism operates even under modest viral loads.

Previous studies using ZIKV-infected human endothelial cells (hcMEC/D3) reported EVs carrying viral components such as RNA, NS1, and E proteins, capable of disrupting endothelial barriers in a BBB model [75]. However, the absence of rigorous purification steps in those experiments leaves open the possibility that co-precipitated virions, rather than EVs alone, mediate these effects. In contrast, our study employed acid glycine and RNase treatments to eliminate residual virions, ensuring that observed outcomes reflect bona fide EVs-mediated viral transfer *in vitro* and *in vivo*.

These findings raise intriguing questions about EVs role in atypical ZIKV transmission and persistence. For instance, sexual transmission documented even post-viremia [76,77], may involve EVs harboring viral RNA in seminal fluid. Prolonged viral RNA detection in immune-privileged sites (e.g., cerebrospinal fluid, semen) could similarly stem from EVs shielding viral genomes from degradation, facilitating viral persistence and tissue-specific pathogenesis. Given the ubiquity of EVs secretion across cell types, their role in disseminating viruses to inaccessible organs like the brain offers critical insights into neurotropism and disease progression [78].

## Conclusions

Our findings demonstrate that EVs derived from ZIKV-infected neonatal cortical neurons and MBECs transport replicative-competent viral genomes, enabling infection initiation within the CNS. This underappreciated dissemination mechanism may contribute to viral persistence and neuropathogenesis by evading early immune detection, infecting distal or less-permissive cells, and establishing viral reservoirs. Elucidating EVs-mediated viral spread provides foundational insights into ZIKV neurovirulence and identifies intercellular communication pathways as potential therapeutic targets to disrupt viral dissemination.

## Supporting information

S1 Fig Infectious virus production by neurons, astrocytes, and MBECs. BHK-21 cells were infected with supernatants collected at various point of time from ZIKV-infected neurons, astrocytes, and MBECs cultures. Neurons and MBECs produced high titters of infectious viral particles as early as 24 hpi, while astrocytes generated detectable infectious particles only from 48 hpi. Representative images from three independent experiments (four replicates per condition, eight fields acquired per well) are shown. Infected cells were quantified using Fiji/ImageJ2 Cell Counter tool, with mean infection percentages indicated above each condition. ZIKV (MOI 1) served as positive infection control, while NIC and supernatants from non-infected cells (Mock) were negative controls. Scale bar: 100 µm.
(TIF)

S2 Fig Western blot characterization of EVs. Cell lysates (NIC and IC) and their corresponding EVs samples (NIC and GlyR) from (A) neurons, (B) astrocytes, and (C) MBECs were separated on 10–12% SDS PAGE gels, transferred to PVDF membranes. EVs canonical markers Alix, Flotillin 1, and CD81 were analyzed, and calreticulin was included as a control. Representative images from two independent experiments with two replicates.
(TIF)

S3 Fig Size distribution profiles of EVs-GlyR. NTA (large panel) and DLS (small panel) demonstrate the size distribution profiles of EVs-GlyR derived from MBEC cultures. Profiles are representative of EVs-GlyR isolated from astrocytes and neurons.
(TIF)

S4 Fig Detection of ZIKV proteins and RNA in EV lysates. A) Western blot analysis of EVs-NIC and EVs-GlyR resolved on 15% SDS–PAGE gels. ZIKV capsid protein (~12 kDa) was detected exclusively in EVs-GlyR from astrocytes (A) and was absent in neuron- (N) or MBEC-derived (EC) EVs-GlyR. ZIKV-infected cell lysate (positive control) and EVs-NIC (negative control) are shown. B) RT-PCR of RNA extracted from EVs lysates (100 ng) yielded ~500 bp ZIKV amplicons only in EVs-IC (untreated with Glycine/RNase). Purified ZIKV RNA and mock controls are included. Gels were stained with ethidium bromide and imaged using a Biorad ChemiDoc ™ system.
(TIF)

S5 Fig Growth and weight changes in EVs-inoculated mice. Mice inoculated with EVs or mock-treated were monitored daily for body size and weight. No significant differences were observed before day 7 post-inoculation. From day 7 onward, ZIKV-infected and EVs-GlyR-treated mice showed significant differences (p < 0.05) compared to Mock and EVs-NIC controls. ZIKV-infected mice exhibited growth arrest and weight loss between days 7–10, while EVsGlyR-treated mice, though smaller than controls, continued gaining weight until the experiment endpoint.
(TIF)

S1 Raw Images All original gels and blots used in this manuscript are provided to ensure full data transparency. (PDF)

## Abbreviations

AA: antibiotic/antimycotic
AraC: cytosine β-D-arabinofuranoside
BBB: Blood-brain barrier
B-CSF: blood–cerebrospinal fluid barrier
C: ZIKV capsid protein
CNS: central nervous system
CPM: count per million
CV: genome coverage
ddPCR: droplet digital PCR
DENV: dengue virus
DLS: Dynamic Light Scattering
E: ZIKV envelope protein
EC: a shorter term for MBEC
EVs: Extracellular vesicles
EVs-EC: MBEC-derived EVs
EVs-IC: EVs from infected cultures
EVs-NIC: EVs from non-infected cultures
EVs-Gly: EVs-IC treated with acid glycine buffer
EVs-GlyR: EVs-IC treated with acid glycine buffer and RNAse
EVs-N: neuron-derived EVs
FBS: fetal bovine serum
Gl Syn: Glutamine synthase
hcMEC/D3: human endothelial cells
HCV: Hepatitis C Virus
HIV: human immunodeficiency virus
Hpi: hours post-infection
HUVEC: human umbilical vein endothelial cells
i.c: intracerebrally
IC: Infected cells
IF: immunofluorescence
JEV: Japanese encephalitis virus
MBECs: mouse brain microvascular endothelial cells
MOI: Multiplicity of infection
N: neurons
NCPM: nucleotide counts per million
NPCs: neural progenitor cells
NTA: Nanoparticle Tracking Analysis
NIC: non-infected controls
Ocln: Occludin
P1: post-natal day 1
PB: Placental barrier
PdI: polydispersity index
PFA: paraformaldehyde
TEM: transmission electron microscopy
UC: ultracentrifugation
ZIKV: Zika virus.
