# Supplementary figures and images for: Neural and endothelial cell-derived extracellular vesicles mediate Zika virus genome dissemination and productive infection in vivo
Source: PLoS One. 2025 Nov 26;20(11):e0337609. doi: 10.1371/journal.pone.0337609 (PMC12654876; doi:10.1371/journal.pone.0337609)

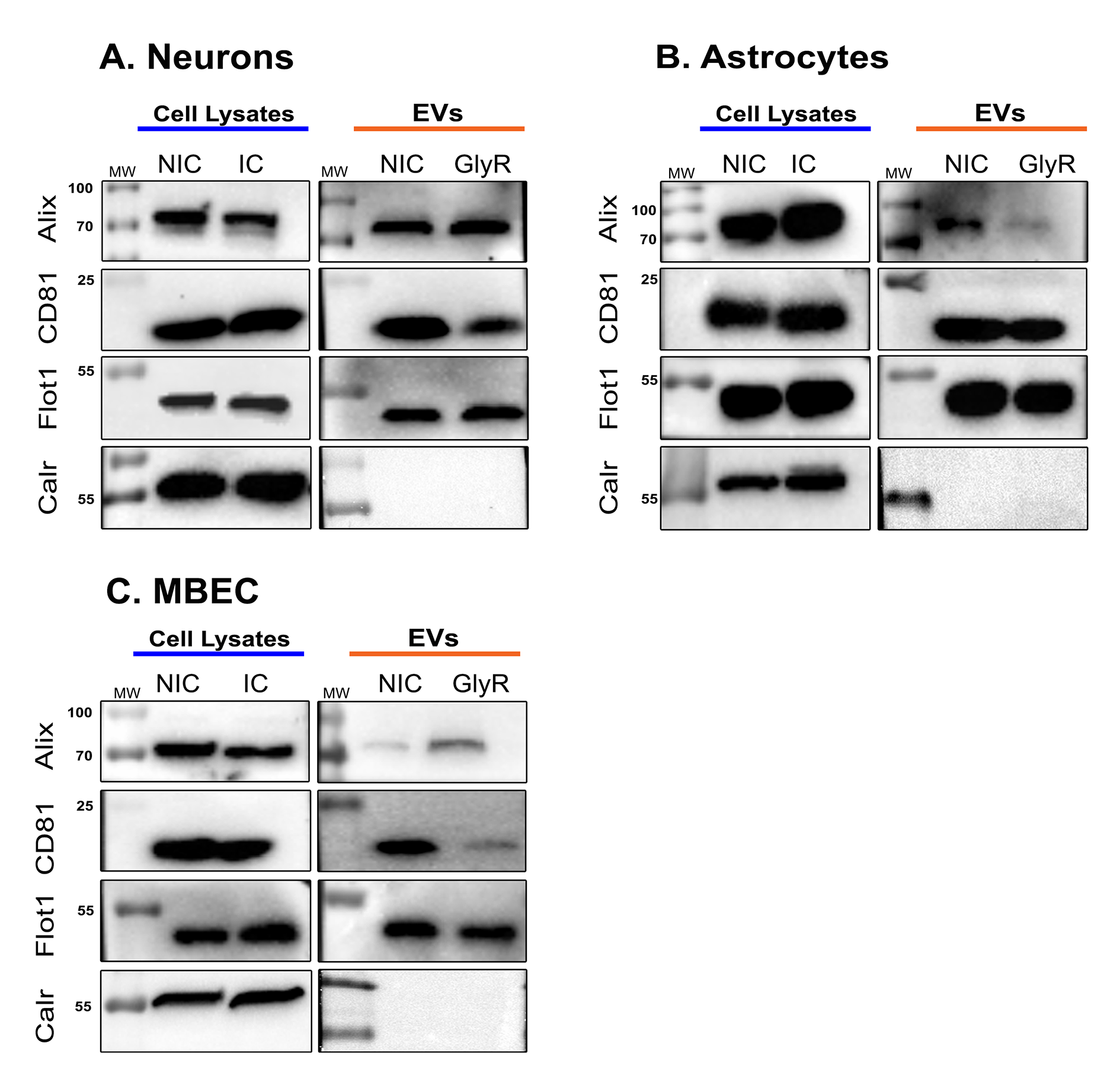

Supplement: S2 Fig — Cell lysates (NIC and IC) and their corresponding EVs samples (NIC and GlyR) from (A) neurons, (B) astrocytes, and (C) MBECs were separated on 10–12% SDS PAGE gels, transferred to PVDF membranes. EVs canonical markers Alix, Flotillin 1, and CD81 were analyzed, and calreticulin was included as a control. Representative images from two independent experiments with two replicates. (TIF) [file pone.0337609.s002.tif]

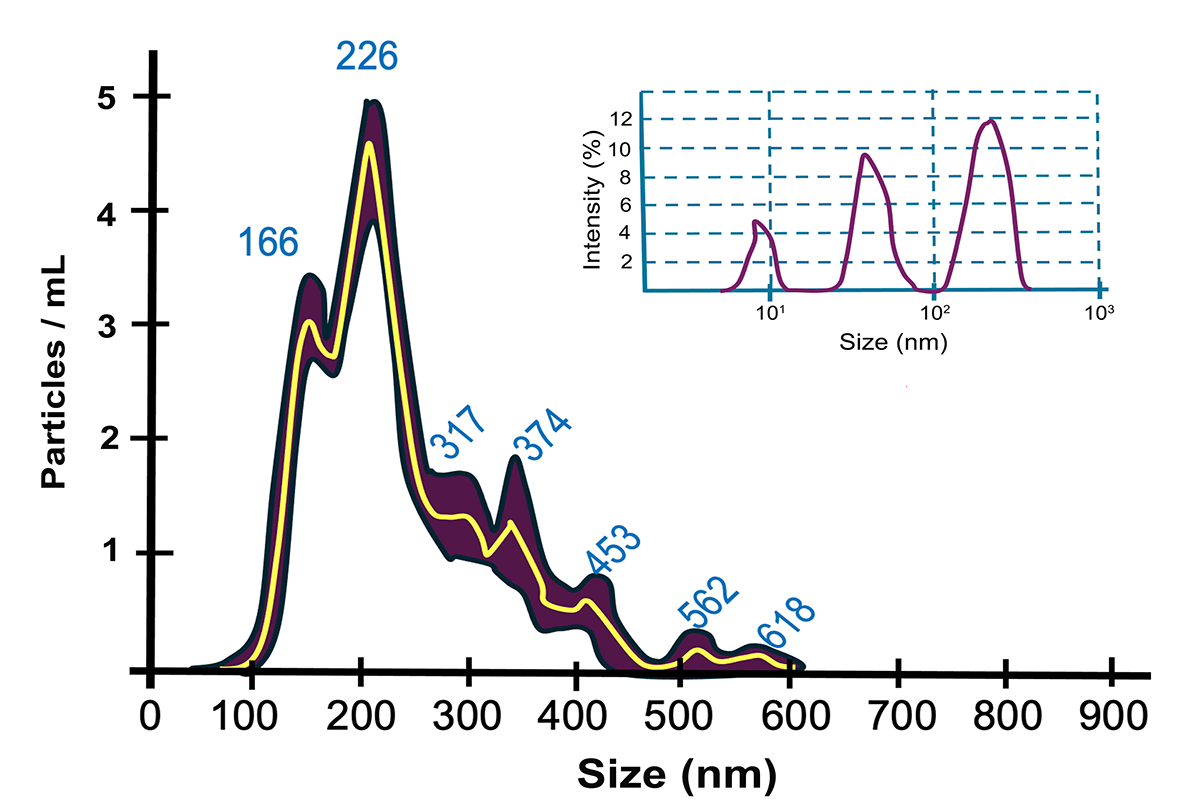

Supplement: S3 Fig — NTA (large panel) and DLS (small panel) demonstrate the size distribution profiles of EVs-GlyR derived from MBEC cultures. Profiles are representative of EVs-GlyR isolated from astrocytes and neurons. (TIF) [file pone.0337609.s003.tif]

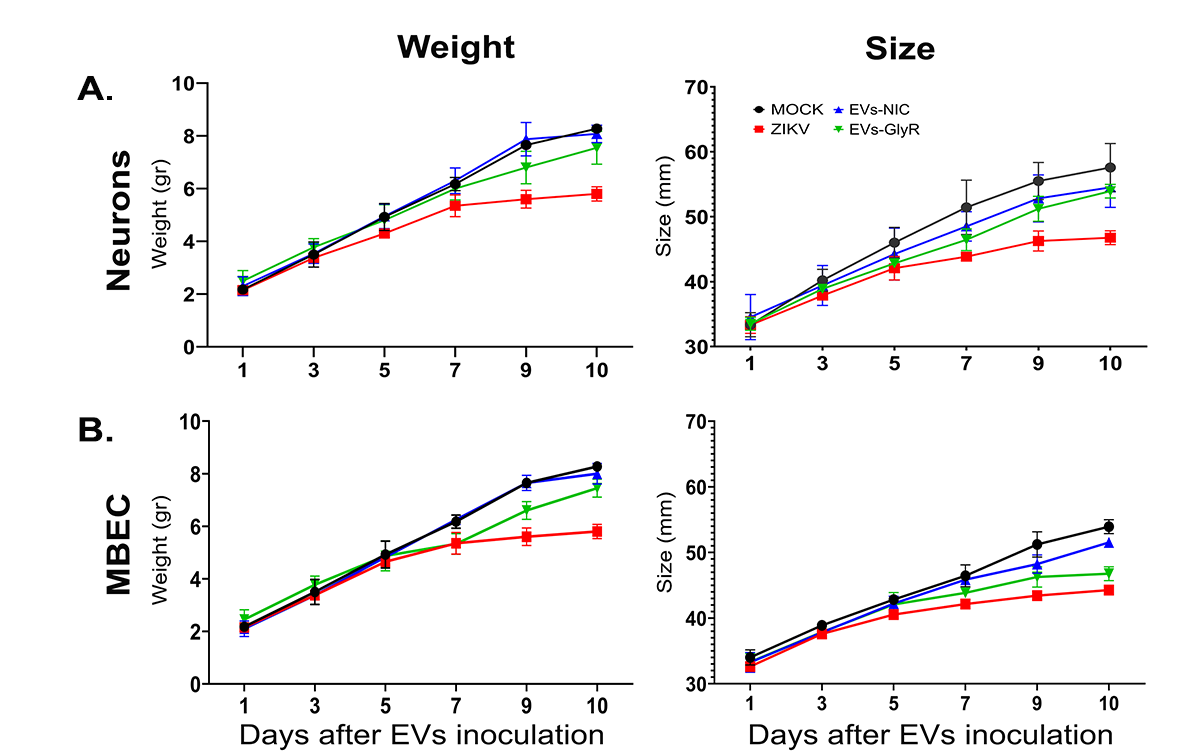

Supplement: S5 Fig — Mice inoculated with EVs or mock-treated were monitored daily for body size and weight. No significant differences were observed before day 7 post-inoculation. From day 7 onward, ZIKV-infected and EVs-GlyR-treated mice showed significant differences (p < 0.05) compared to Mock and EVs-NIC controls. ZIKV-infected mice exhibited growth arrest and weight loss between days 7–10, while EVsGlyR-treated mice, though smaller than controls, continued gaining weight until the experiment endpoint. (TIF) [file pone.0337609.s005.tif]
