# Supplementary material for: Neural and endothelial cell-derived extracellular vesicles mediate Zika virus genome dissemination and productive infection in vivo
Source: PLoS One. 2025 Nov 26;20(11):e0337609. doi: 10.1371/journal.pone.0337609 (PMC12654876; doi:10.1371/journal.pone.0337609)

This image corresponds to Figure 8A.
WB to Capsid and β-actin in whole brain lysates
inoculated with different EVs: Neurons (N) or endothelial cells (EC).
Positive control: Animals inoculated with Zika virus
Negative control: Animals exposed to C6/36 supernatants (Mock)
or inoculated with EVs from uninfected cells (NIC).

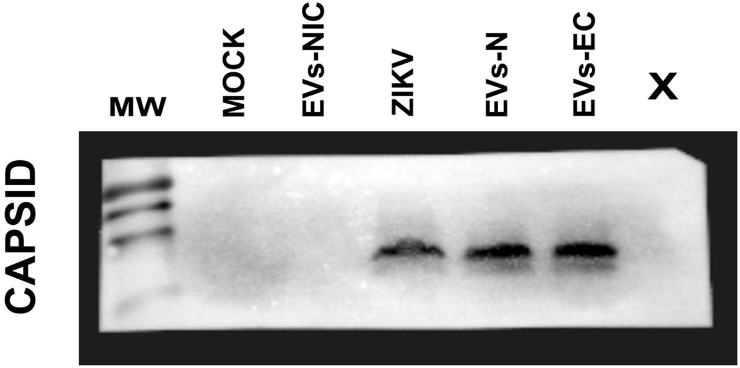

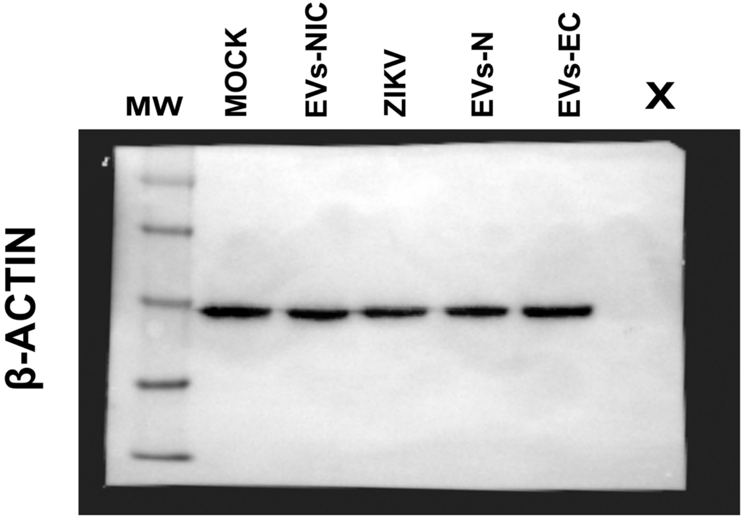

This image corresponds to Figure Supple 4A.
WB for capsid in EVs from neurons, astrocytes,
or endothelial cells, treated with buffer glycine and RNase A,
compared with EVs from uninfected cells (NIC).
Positive control: Vero cells infected with ZIKV or
cells exposed to C6/36 supernatants (Mock).

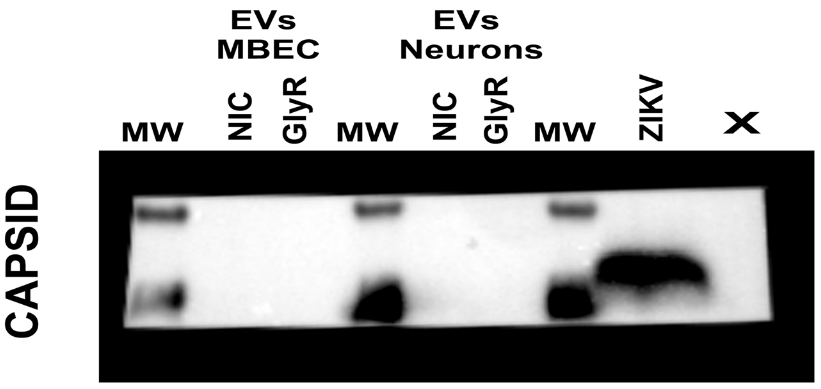

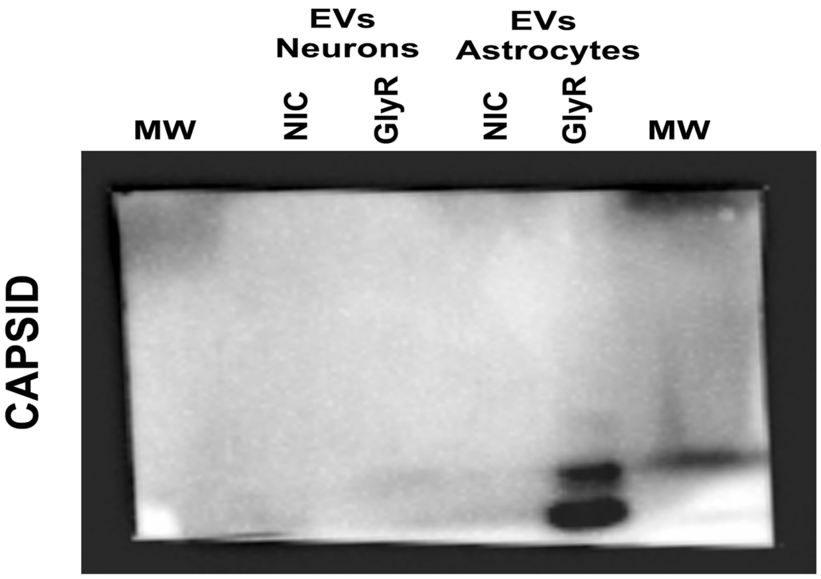

This image corresponds to Figure Supple 4B.
Agarose gel with PCR products amplifying the complete ZIKV genome
present in EVs from different cells treated with buffer glycine and RNase A.
Two different primer sets were used to amplify the complete genome.
Positive control: ZIKV RNA used in the model.
Mock supernatant from C6/36.

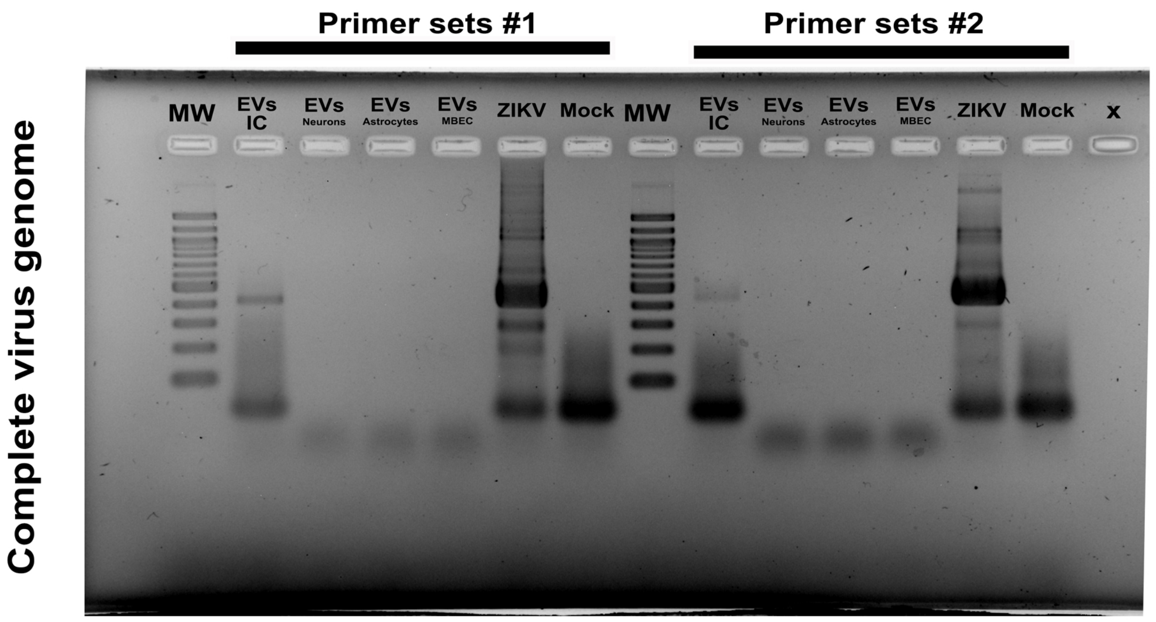

Supplement: S1 Raw Images — (PDF) [file pone.0337609.s006.pdf]
